# Adipose-Derived Stem Cells from Obese Donors Polarize Macrophages and Microglia toward a Pro-Inflammatory Phenotype

**DOI:** 10.3390/cells10010026

**Published:** 2020-12-25

**Authors:** Mark A. A. Harrison, Rachel M. Wise, Brooke P. Benjamin, Emily M. Hochreiner, Omair A. Mohiuddin, Bruce A. Bunnell

**Affiliations:** 1 Neuroscience Program, Tulane Brain Institute, Tulane University School of Science & Engineering, New Orleans, LA 70118, USA; mharri26@tulane.edu (M.A.A.H.); rwise@tulane.edu (R.M.W.); ehochreiner@tulane.edu (E.M.H.); 2 Center for Stem Cell Research & Regenerative Medicine, Tulane University School of Medicine, New Orleans, LA 70112, USA; bbenjamin@tulane.edu (B.P.B.); omairmohiuddin1@gmail.com (O.A.M.); 3 Dr. Panjwani Center for Molecular Medicine and Drug Research, International Center for Chemical and Biological Science, University of Karachi, Karachi 75270, Pakistan; 4 Department of Microbiology, Immunology and Genetics, University of North Texas Health Science Center, Fort Worth, TX 76107, USA; 5 Department of Pharmacology, Tulane University School of Medicine, New Orleans, LA 70112, USA

**Keywords:** adipose tissue, adipose stem cells (ASCs), obesity, polarization, macrophage, microglia, immunomodulation, inflammation

## Abstract

Macrophages and microglia represent the primary phagocytes and first line of defense in the peripheral and central immune systems. They activate and polarize into a spectrum of pro- and anti-inflammatory phenotypes in response to various stimuli. This activation is tightly regulated to balance the appropriate immune response with tissue repair and homeostasis. Disruption of this balance results in inflammatory disease states and tissue damage. Adipose stem cells (ASCs) have great therapeutic potential because of the potent immunomodulatory capabilities which induce the polarization of microglia and macrophages to the anti-inflammatory, M2, phenotype. In this study, we examined the effects of donor heterogeneity on ASC function. Specifically, we investigated the impact of donor obesity on ASC stemness and immunomodulatory abilities. Our findings revealed that ASCs from obese donors (ObASCs) exhibited reduced stem cell characteristics when compared to ASCs from lean donors (LnASCs). We also found that ObASCs promote a pro-inflammatory phenotype in murine macrophage and microglial cells, as indicated by the upregulated expression of pro-inflammatory genes, increased nitric oxide pathway activity, and impaired phagocytosis and migration. These findings highlight the importance of considering individual donor characteristics such as obesity when selecting donors and cells for use in ASC therapeutic applications and regenerative medicine.

## 1. Introduction

Adipose tissue-derived stem cells (ASCs), a type of mesenchymal stem cell (MSC), possess significant anti-inflammatory and immunomodulatory properties that make them an attractive therapeutic option for numerous inflammatory diseases. ASCs exert their effects on the adaptive and innate immune system via the secretion of soluble molecules and extracellular vesicles (EVs), which alter the inflammatory microenvironment [1,2,3,4]. This is partially accomplished by transitioning activated macrophages from pro-inflammatory toward anti-inflammatory, pro-repair phenotypes [1,2]. Additionally, pre-treatment of ASCs with pro-inflammatory factors or hypoxic conditions strengthens their immunomodulatory abilities [3,5,6,7,8]. These stressful conditions mimic the post-transplant tissue niche and provide some evidence for their behavior in vivo. Because of these properties, ASCs have been investigated as therapeutic agents in animal models of various diseases such as multiple sclerosis (MS) [9,10], uveitis [11,12], kidney injury [13,14], inflammatory bowel disease [15,16,17], and cutaneous wound healing [18,19]. Results from these studies suggest that ASCs possess robust immunosuppressive potential in numerous disease states; however, the application of ASC-based therapies to the clinic has met with limited success.

One hindrance to clinical translation may be the heterogeneity of ASC function among different donors. It is known that individual donor characteristics such as age and comorbidities can alter the physiology and function of ASCs [20,21,22,23,24,25,26,27]. Research has shown that generating pools of donor ASCs can mitigate some donor-to-donor variation and may help combat these effects [28]. However, the examination of donor characteristics and their impact on ASC function represents a valuable area of research that will inform donor selection and prove important for future clinical trials. Specifically, the aim of this study is to examine the impact of exposure to ASCs from either lean or obese donor pools on circulating or tissue resident phagocytes. An investigation of phenotypic and functional changes in these phagocytes will provide valuable insight into the differential effects of ASCs dependent on obesity status.

Obesity is characterized by increased deposition of white adipose tissue in both the subcutaneous and visceral fat depots. This increase in adipose tissue is a result of both hypertrophy, an increase in adipocyte size, and hyperplasia, an increase in adipocyte proliferation [29]. Obesity-related adipose tissue hypoxia also causes the activation of signaling cascades that lead hypertrophic adipocytes to express pro-inflammatory cytokines [30,31]. These cytokines activate tissue-resident macrophages and circulating lymphocytes, leading to a chronic state of tissue inflammation [32]. In response to the chronic inflammation and hypoxia in obese adipose tissue, ASCs exhibit distinct physiological changes. Studies have demonstrated that ASCs from obese individuals (ObASCs) produced elevated levels of pro-inflammatory mediators [33,34,35], exhibit reduced stem cell characteristics [33,34,36,37,38], and display reduced immunomodulatory abilities [39]. Our laboratory has previously shown that ObASCs do not possess the same therapeutic efficacy as lean ASCs (LnASCs) in a mouse model of MS [26]. We also showed that ObASCs produce an exaggerated immune response following exposure to an inflammatory environment, which we suspect resulted in increased CD4^+^ and CD8^+^ T-cell proliferation and the increased lesion area seen in the mouse model of MS [26]. These findings are intriguing; however, the effects of ObASCs on phagocytes of the innate immune system have not yet been elucidated.

Macrophages and microglia, brain-resident macrophages, are primary components of the innate immune system and represent the first line of defense against foreign pathogens and tissue damage. In response to an array of extracellular signals, they activate and adopt phenotypically and functionally distinct profiles. Initially, these were described as either classically activated (M1), pro-inflammatory, or alternatively activated (M2), anti-inflammatory phenotypes [40,41]. More recently, however, the spectrum of macrophages has been expanded from this binary system to one which more accurately encompasses the diverse phenotypic and functional behavior of macrophages [42]. Appropriate pro-inflammatory macrophage and microglial response is essential for defense against pathogens however, if left unchecked it can result in significant tissue damage. Therefore, induction of anti-inflammatory, pro-repair, and regulatory macrophages are essential for orchestration of the adaptive immune response, resolution of inflammation, and initiation of tissue repair [41,43].

Considering the importance of macrophage polarization and function following tissue damage or infection, this study aimed to determine whether obesity status of ASC donors alters the phenotypic and functional characteristics of these highly mutable cells. We hypothesized that ObASCs have been fundamentally altered by their chronically inflamed tissue niche and that this results in an impaired ability to promote an anti-inflammatory phenotype in murine macrophages and microglia. To investigate this, we compared standard stem cell characteristics of LnASCs and ObASCs. Next, we determined the effects of indirect co-culture of LnASCs and ObASCs on both macrophages and microglia. Our results provide additional evidence for the impact of obesity on ASC stemness, relate these changes to the immunomodulatory capabilities of ASCs, and provide mechanistic insight into previous findings that indicate a loss of therapeutic efficacy in an animal model of inflammatory disease [26].

## 2. Materials and Methods

### 2.1. Cell Culture

Human female, subcutaneous, abdominal ASCs were obtained from LaCell LLC/Obatala Sciences Inc. (New Orleans, LA, USA). Individual ASC cell lines were previously fully characterized by our laboratory prior to being pooled [25,26,44,45,46]. Following pooling an abbreviated characterization was completed to ensure expression of basic stemness characteristics. Lean donors were considered those with a BMI less than 25 kg/m^2^ while obese donors were considered those with BMI greater than 30 kg/m^2^ as previously described by Sabol et al. 2019 [45]. Donor pools were created using lean donors (LnASCs; n = 6; BMI = 22.45 ± 1.51; age = 36.33 ± 5.62) and obese donors (ObASCs; n = 6; BMI = 33.97 ± 3.11; age = 40.50 ± 7.46). Cells were maintained in stromal media consisting of Dulbecco′s modified Eagle′s media and nutrient mixture F12 (DMEM: F12; ThermoFisher; Waltham, MA, USA) supplemented with 10% heat-inactivated fetal bovine serum (FBS; Hyclone; Logan, UT, USA) and 1% anti-mycotic, anti-biotic (ThermoFisher). Cells were harvested at passage four (p4) with 0.25% trypsin, 1 mM EDTA (ThermoFisher) and all experiments were conducted with p5 ASCs. As in previous studies, immortalized macrophage and microglial cell lines were used in order to control the passage number and to avoid the impact of accidentally co-isolated cell populations common in primary culture [47,48,49,50]. The murine microglial cell line SIM-A9 was purchased from ATCC (Manassas, VA, USA) and maintained in DMEM: F12 culture medium supplemented with 10% heat-inactivated FBS (ThermoFisher), 5% heat inactivated horse serum (HS; ThermoFisher), and 1% anti-mycotic, anti-biotic. SIM-A9 cells were lifted for passage by incubating with 1xPBS supplemented with 1 mM EGTA (Sigma, St. Louis, MO, USA), 1 mM EDTA (Sigma), and 1 mg/mL glucose (Sigma). The murine monocyte/macrophage cell line RAW264.7 was also purchased from ATCC and was cultured in high-glucose DMEM (ThermoFisher) supplemented with 10% FBS and 1% anti-mycotic, anti-biotic. RAW264.7 cells were lifted for passage by manual scraping when cells reached 80% confluence. All murine lines were used between passage 5 and 10.

### 2.2. Characterization of ASCs

*Flow Cytometry:* Phenotypic characterization of pooled human ASCs was completed using flow cytometric analysis of positive and negative surface markers as previously described [51,52]. Briefly, cells were harvested, washed with 1xPBS, and incubated with fluorochrome-conjugated primary antibodies at room temperature (RT) for 15 min. Cells were then fixed in 1% paraformaldehyde (PFA) (Santa Cruz Biotechnology; Dallas, TX, USA) for 5 min at RT and analyzed with a Gallios Flow Cytometer and Kaluza software (Beckman Coulter; Brea, CA, USA). The following antibodies were purchased from BD Biosciences (San Jose, CA, USA): anti-CD3-PE-Texas Red, anti-CD31 PE-Cy7, and anti-CD73-PE. Anti-CD90-FITC and anti-CD105-APC were purchased from Invitrogen (Waltham, MA, USA). Finally, anti-CD14-PECy5 and anti-CD45-AF700 were purchased from Beckman Coulter.

*Adipogenic Differentiation and Quantification:* ASCs were seeded at 1 × 10^5^ cells per well in a 12-well plate (Corning Inc.; Corning, NY, USA) and cultured in stromal media until confluent. Cells were then switched into adipocyte differentiation media (ADM) consisting of stromal media supplemented with dexamethasone (1 μM; Sigma), isobutylmethylxanthine (IBMX) (250 μM; Sigma), rosiglitazone (5 μM; Sigma), biotin (66 μM, Cayman Chemical; Ann Arbor, MI, USA), calcium d-pantothenate (34 μM, Sigma), and human insulin (200 nM, Sigma). ASCs were maintained in ADM for 21 days, with fresh ADM or adipose maintenance media (ADM without IBMX and rosiglitazone) added every 3 days on an alternating cycle. After 21 days cells were fixed for 30 min with 4% PFA (Santa Cruz) followed by incubation in a 0.5% solution of Oil-Red-O (Sigma)for 10 min at RT. Neutral lipid droplets were imaged with a 10× objective on a Nikon Eclipse TE-200 (Nikon; Melville, NY, USA) using Nikon′s ACT-1 software. Quantification of Oil-Red-O staining was accomplished by destaining with 100% isopropanol and absorbance at 584 nm was determined using a Synergy HTX plate reader (BioTek; Winooski, VT, USA). Differentiation was reported as a percentage of control well staining.

*Colony Forming Unit Fibroblast (CFU-F) Assay:* ASCs were seeded at 5 × 10^2^ cells per 10 cm plate (Corning Inc.) and cultured for 14 days. On day 14, cells were fixed and stained with 3% crystal violet (Sigma) in methanol (Sigma) for 30 min at RT. Plates were then washed with DI water until clear and the number of colonies with a diameter greater than 2 mm was manually recorded.

*Population Doubling Time:* ASCs were seeded at 1 × 10^4^ cells per well in a 6-well plate and cultured in stromal media. Every 24 h for a total of 8 days cells were harvested with 0.25% trypsin and 1 mM EDTA (ThermoFisher), and viable cells were manually counted using trypan blue exclusion (ThermoFisher). Population doubling times (*DT*) were calculated using the previously described Equation [53]:

(1) $$DT=\frac{CT\times ln\;2}{ln\;\left(Nf/Ni\right)}$$

where *CT* is culture time in hours, *Ni* is the initial cell number as counted on day 1, and *Nf* is the final cell number. Doubling time was reported as the mean ± standard deviation.

### 2.3. Indirect Co-Culture Experiments

*Co-Culture Conditions:* Indirect co-culture of RAW264.7 or SIM-A9 cells with ASCs was accomplished using polyethylene terephthalate (PET) Transwells with a diameter of 24 mm and a pore size of 0.4 μm (Corning Inc.). ASCs were seeded at 5 × 10^4^ cells per Transwell in stromal media. ASCs were treated with human interferon gamma (hIFNγ; 20 ng/mL; EMD Millipore; Billerica, MA, USA) for 48 h to activate them and enhance their immunomodulatory activity as previously described [3,6]. Concurrently, RAW264.7 or SIM-A9 were seeded into 6-well plates at 5 × 10^4^ or 2 × 10^4^ cells per well, respectively. After 48 h, all cells were rinsed with 1xPBS, fresh media was replaced, and ASC seeded Transwell inserts were moved into the 6-well plate for a 48-h co-culture. After 48 h, inserts containing ASCs were removed and RAW264.7 or SIM-A9 cells were imaged for morphology, lysed for RNA isolation, or maintained for a further 48 h in culture to generate conditioned medium. Control wells with no ASCs were run in parallel. All experiments were performed in triplicate.

*RNA Isolation and qRT-PCR:* RNA was isolated from RAW264.7 and SIM-A9 cells using a RNeasy Mini Kit (Qiagen). Following RNA isolation, 1 μg of total mRNA was used to synthesize cDNA using the Applied Biosystems High-Capacity cDNA Reverse Transcription Kit (ThermoFisher). Quantitative reverse transcription PCR (qRT-PCR) was performed using SsoAdvanced Universal SYBR Green SuperMix (Bio-Rad; Hercules, CA, USA) according to the manufacturer′s instructions. Oligonucleotide primer sets were designed using PrimerBLAST software and manufactured by Integrated DNA Technologies (IDT; Coralville, IA, USA) and sequences are listed in Table 1. Relative gene expression was determined using the 2^−ΔΔCt^ method and reported as fold change relative to untreated controls following normalization to the housekeeping gene, 40 S ribosomal protein S29 (RPS29).

*Migration Assay:* Migration efficiency of the RAW264.7 macrophages and SIM-A9 microglia in the presence or absence of LnASCs or ObASCs was determined using Transwells with a diameter of 6.5 mm and a pore size of 5.0 μm (Corning Inc.). Transwells were coated with rat tail collagen type 1 (Corning) at a concentration of 50 μg/mL for 2 h at 37 °C, then dried and stored at 4 °C until use. Meanwhile, LnASCs and ObASCs were seeded into 24-well plates at 1 × 10^4^ cells per well and pre-stimulated for 24 h with hIFNγ as described above. ASCs were then washed with 1xPBS and stromal media was replaced with serum-free media. Transwell inserts were rehydrated with serum-free media for 30 min and RAW264.7 or SIM-A9 cells were seeded at 1 × 10^5^ or 5 × 10^4^ cells per Transwell, respectively, in serum-free media. RAW264.7 macrophages were allowed to migrate for 72 h, and SIM-A9 microglia were allowed to migrate for 24 h. After migration, Transwells were removed and stained with 3% crystal violet (Sigma) in methanol for 30 min at RT. Transwells were washed with DI water and the upper surface of the Transwell was brushed with a cotton swab to remove non-migrated cells. As previously reported by Yin et al. 2017 and Yu et al. 2018, three random fields were chosen and imaged using a 10× objective for each Transwell [54,55]. The number of migrated cells was manually counted for each image by a group-blinded researcher and reported as the average cells migrated per field.

*Phagocytosis Assay:* RAW264.7 and SIM-A9 cells were seeded separately into 96-well plates at 2.5 × 10^3^ cells per well in either control growth media or media supplemented with 50% LnASC or ObASC conditioned media and cultured for 48 h. The CytoSelect Phagocytosis Assay *E. coli* Substrate Kit (Cell Biolabs; San Diego, CA, USA) was employed to assess phagocytic ability according to the manufacturer’s instructions. Briefly, cells were incubated with *E. coli* for 4 h at 37 °C, followed by fixation, permeabilization, and incubation with substrate to visualize phagocytosis. Absorbance was read at 450 nm on a Synergy HTX plate reader (BioTek).

*Nitric Oxide Production:* After co-culture, RAW264.7 or SIM-A9 cells were cultured for a further 48 h and conditioned media (CM) was collected and immediately frozen at −80 °C. When thawed, CM was diluted 1:2 and a Griess assay was used to determine the levels of NO metabolites according to the manufacturer’s instructions (Cell Signaling Technology; Danvers, MA, USA). Briefly, samples were run in triplicate, incubated in sulfanilamide solution for 10 min at RT, and absorbance was measured at 540 nm with a wavelength correction at 690 nm using a BioTek Synergy HTX plate reader. Nitrite concentrations in CM were extrapolated from a standard curve.

### 2.4. Statistical Analysis

All data are expressed as mean ± standard deviation from at least three independent experiments. The statistical differences between two groups was determined by a Mann-Whitney test. Differences between three or more groups was determined by one-way ANOVA followed by Dunnett’s multiple comparisons test. For all comparisons, a *p* < 0.05 was considered to indicate a significant difference. GraphPad Prism 8 software was used for all statistical analyses (GraphPad; San Diego, CA, USA).

## 3. Results

### 3.1. ObASCs Exhibit Reduced Stemness Characteristics When Compared with LnASCs

**Figure 1:**
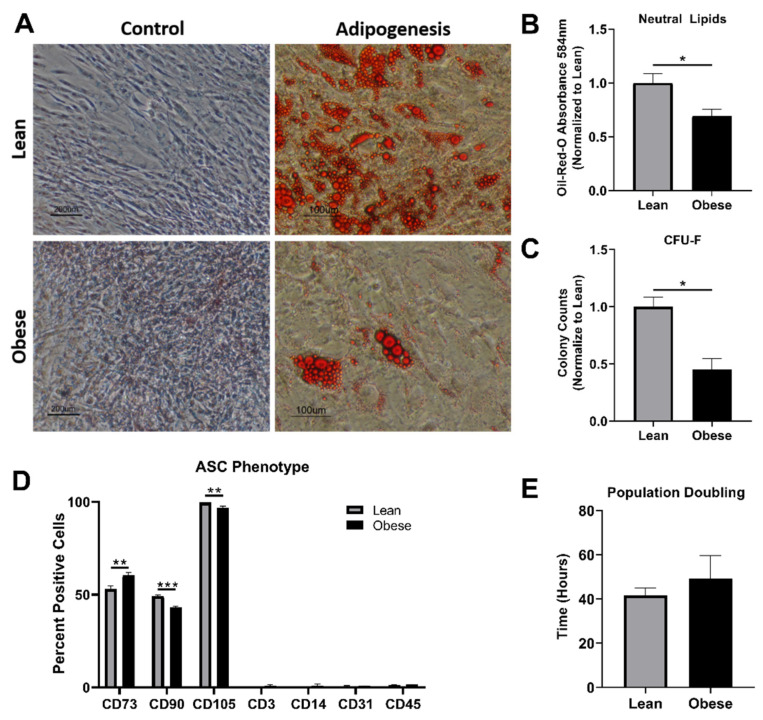
Pooled ObASCs exhibit reduced stemness characteristics. (**A**) LnASCs and ObASCs were differentiated with adipogenic media for 21 days and stained with Oil-Red-O and imaged at 10× (scale bar is 200 μm for controls and 100 μm for adipogenesis). (**B**) Oil-Red-O destaining absorbance was measured at 584 nm and normalized to the LnASCs. (**C**) CFU-F assay was completed with both LnASCs and ObASCs and the results were normalized to the LnASC colony counts. (**D**) Flow cytometry analysis of LnASCs and ObASCs using both positive and negative MSC markers. (**E**) Average population doubling time of LnASCs and ObASCs expressed in hours. Values are presented as means (N = 3) ± SD of three independent experiments using Mann-Whitney tests. Statistical differences between the means are marked with * *p* < 0.05, ** *p* < 0.01, *** *p* < 0.001. Abbreviations: LnASCs, lean ASCs; ObASCs obese ASCs; CD, cluster of differentiation.

Human adipose stem cells (ASCs) were pooled from six lean (BMI < 25) or six obese (BMI > 30) donors. No statistical differences were found in the age of donors between groups (*p* = 0.513). Similar cell morphology was seen in both LnASCs and ObASCs (data not shown). Following a 21-day adipogenic differentiation, both LnASCs and ObASCs differentiated into adipocytes, which produced lipid droplets (Figure 1A). However, the degree of adipogenic differentiation observed in ObASCs was significantly lower than that of LnASCs (Figure 1B). Colony-forming unit fibroblast (CFU-F) assay indicated that ObASCs had an impaired ability to form colonies relative to LnASCs (Figure 1C). Flow cytometric analysis of the ASC phenotype based on positive (CD73, CD90, CD105) and negative (CD4, CD14, CD31, CD45) markers was conducted for both groups (Figure 1D). The data indicated that the ObASCs exhibited significantly decreased expression of CD90 and CD105 relative to LnASCs. Interestingly, ObASCs exhibited a slightly elevated level of CD73 relative to LnASCs (*p* < 0.01). Finally, the average population doubling time was determined for each pool over an 8-day period and revealed no significant differences between groups (Figure 1E).

### 3.2. Indirect Co-Culture with ObASCs, But Not LnASCs, Induces Polarization toward M1 Phenotype in RAW264.7 Macrophages

**Figure 2:**
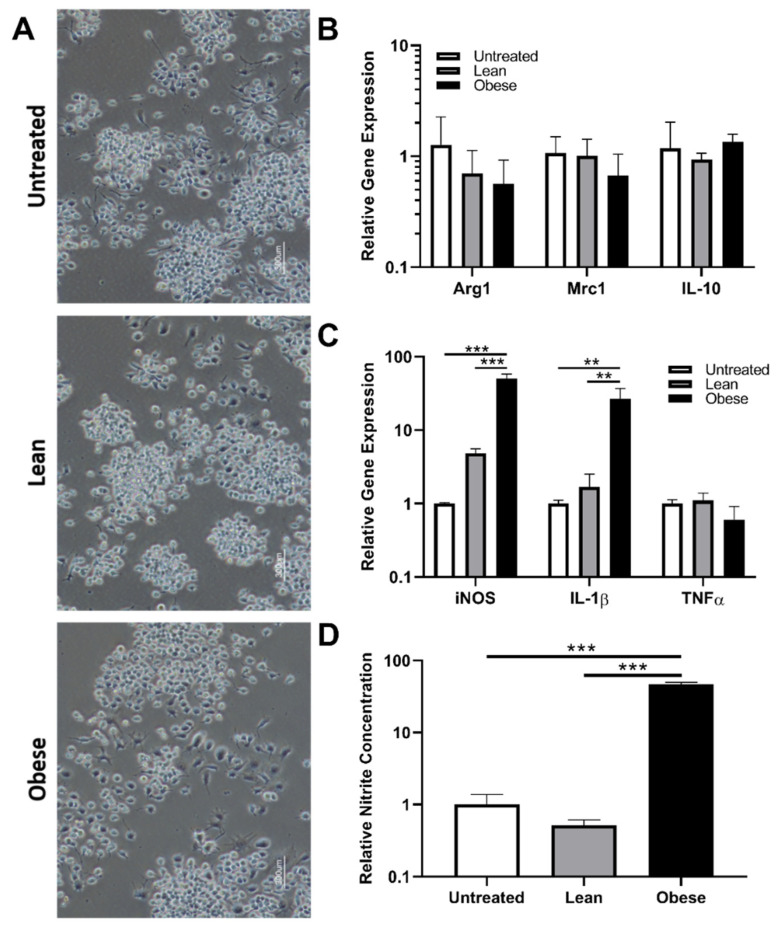
RAW264.7 cells following indirect co-culture with ASCs in a Transwell system. (**A**) Representative images of RAW264.7 morphology following either LnASC or ObASC co-culture for 48-h. (**B**) Changes in anti-inflammatory gene expression of RAW264.7 cells following 48-h co-culture relative to untreated controls. (**C**) Changes in pro-inflammatory gene expression of RAW264.7 cells following 48-h co-culture relative to untreated controls. (**D**) Griess assay determination of nitrite concentration of 48-h conditioned media following 48-h co-culture relative to untreated controls. Values are presented as means (N = 3) ± SD of three independent experiments using one-way ANOVA followed by Dunnett’s multiple comparisons test. Statistical differences between the means are marked with ** *p* < 0.01, *** *p* < 0.001. Abbreviations: Arg1, arginase 1; Mrc1, mannose receptor C-type 1; IL-10, interleukin 10; iNOS, inducible nitric oxide synthase; IL-1β, interleukin 1 beta; TNFα, tumor necrosis factor alpha.

To determine the effect of LnASCs’ and ObASCs’ secretome on macrophage gene expression and function, indirect co-culture with RAW264.7 cells for 48 h was performed. The morphology of RAW264.7 cells following co-culture was examined and no significant alterations were noted between groups (Figure 2A). The gene expression analysis of key anti-inflammatory (Figure 2B) factors revealed no significant changes in anti-inflammatory gene expression following either LnASC or ObASC co-culture relative to untreated macrophages. However, co-culture with ObASCs resulted in a roughly 50-fold increase in expression of inducible nitric oxide synthase (iNOS) and a 26-fold increase in interleukine-1 beta (IL-1β) expression (Figure 2C). Similarly, in comparison to LnASCs, ObASCs induced a 10-fold and a 16-fold increase in iNOS and IL-1β expression respectively. Consistent with the upregulation of iNOS transcripts, RAW264.7 cells co-cultured with ObASCs demonstrated a 47-fold increase in iNOS metabolite production relative to untreated control cells as measured by a Griess assay (Figure 2D). These findings were echoed in the 90-fold increase in iNOS metabolites in ObASC exposed cells relative to LnASC exposed cells.

### 3.3. LnASCs and ObASCs Differentially Affect the Migration and Phagocytic Abilities of RAW264.7 Macrophages

**Figure 3:**
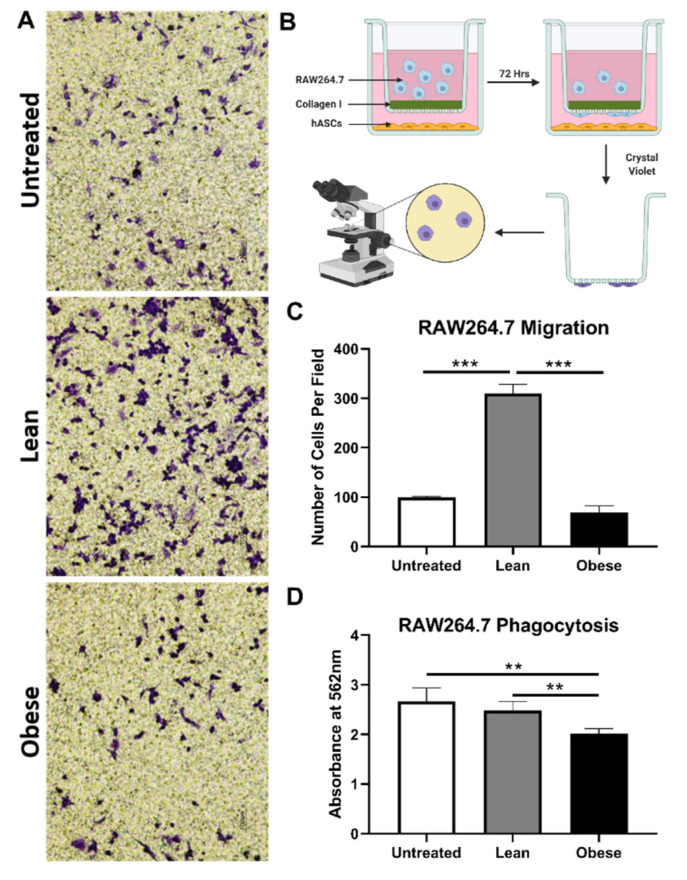
LnASCs and ObASCs differentially alter the migration and phagocytotic abilities of RAW264.7 cells. (**A**) Representative scheme of RAW264. 7 cells following migration through a collagen-coated Transwell in the presence of LnASCs or ObASCs. Created with Biorender.com (**B**) Diagram of migration assay process including staining and imaging. (**C**) Quantification of the cells migrated per field based on three randomly chosen fields for each Transwell migration performed. (**D**) Phagocytosis of *E. coli*-substrate by RAW264.7 cells following 48-h treatment with LnASC or ObASC conditioned media. Values are presented as means (N = 3) ± SD of three independent experiments using one-way ANOVA followed by Dunnett’s multiple comparison test. Statistical differences between the means are marked with ** *p* < 0.01, *** *p* < 0.001.

Following the changes in gene expression observed in RAW264.7 cells, we sought to determine what impact LnASCs and ObASCs have on the functional properties of macrophages, namely migration and phagocytosis. The number of macrophages able to digest the collagen-coating and migrate through a 0.4μm-pore Transwell toward the ASCs was used to determine their migratory abilities. Following 72-h co-culture, more than three times as many RAW264.7 cells migrated through the collagen-coated Transwell when cultured with LnASCs than untreated controls (*p* < 0.001) (Figure 3C). Likewise, a comparison between LnASC and ObASC exposed groups resulted in more than four times as many RAW264.7 cells migrated. There was no statistical difference between ObASC co-cultured RAW264.7 cell migration and untreated control cells. The ability of RAW264.7 macrophages to phagocytose when cultured with LnASC or ObASC conditioned media (CM) was assessed with colorimetric quantitative analysis of engulfed *E. coli* particles. When cultured with ObASC CM, but not LnASC CM, macrophages exhibited a 24% decrease in phagocytic ability when compared to untreated control macrophages (Figure 3D). The decrease in phagocytic ability between LnASC and ObASC groups was less extreme at a 20% decrease, but still statistically significant.

### 3.4. Indirect Co-Culture with ObASCs, But Not LnASCs, Induces Polarization toward M1 Phenotype in SIM-A9 Microglia

**Figure 4:**
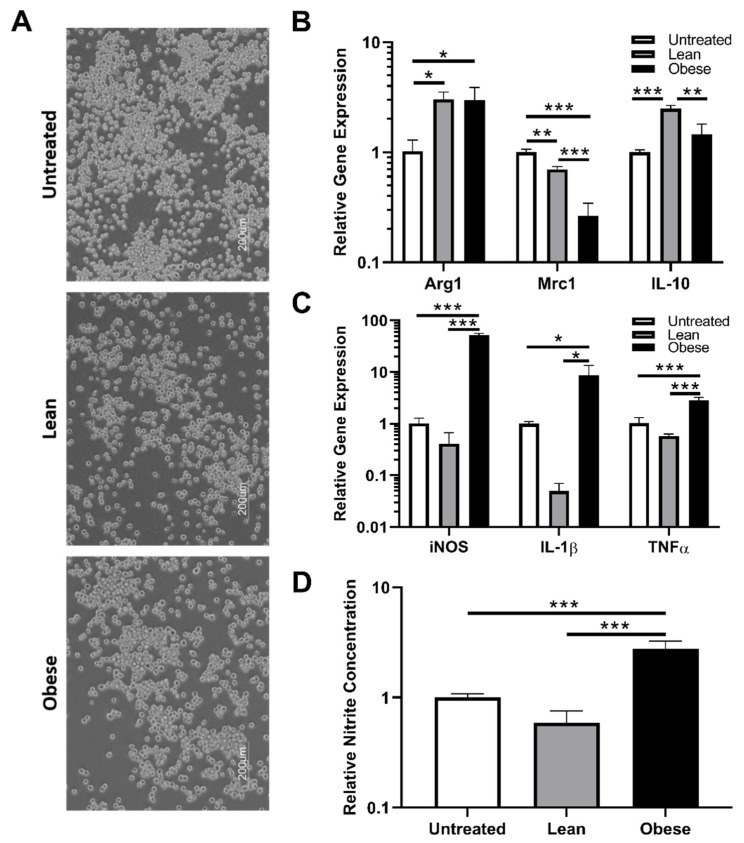
SIM-A9 cells following indirect co-culture with ASCs in a Transwell system. (**A**) Representative images of SIM-A9 morphology following either LnASC or ObASC co-culture. (**B**) Changes in anti-inflammatory gene expression of SIM-A9 cells following 48-h co-culture relative to untreated controls. (**C**) Changes in pro-inflammatory gene expression of SIM-A9 cells following 48-h co-culture relative to untreated controls. (**D**) Griess assay determination of nitrite concentration of 48-h conditioned media following 48-h co-culture relative to untreated cells. Values are presented as means (N = 3) ± SD of three independent experiments using one-way ANOVA followed by Dunnett’s multiple comparison test. Statistical differences between the means are marked with * *p* < 0.05, ** *p* < 0.01, *** *p* < 0.001. Abbreviations: Arg1, arginase 1; Mrc1, mannose receptor C-type 1; IL-10, interleukin 10; iNOS, inducible nitric oxide synthase; IL-1β, interleukin 1 beta; TNFα, tumor necrosis factor alpha.

Microglia are the primary brain-resident macrophages and represent the second line of CNS defense following the blood-brain barrier. Since we observed significant changes in macrophage physiology and function, the impacts of LnASC and ObASCs on microglia were assessed to determine how consistent these effects are on cells of similar functionality but differing origin. The morphology of SIM-A9 cells was consistent across all co-cultures (Figure 4A). Following 48-h co-culture, alterations in gene expression of anti-inflammatory (Figure 4B) and pro-inflammatory (Figure 4C) factors were examined in LnASC and ObASC co-culture samples relative to untreated controls. Interestingly, both LnASC and ObASC co-culture enhanced the expression of arginase-1 (Arg1) but lacked differences of expression when compared with each other. However, another anti-inflammatory marker, mannose receptor 1 (Mrc1; CD206) was downregulated to 0.7-fold following LnASC co-culture. This effect was further decreased to 0.26-fold expression following ObASC co-culture and exhibited a statistically significant decrease when compared with the LnASC group. Importantly, expression of the anti-inflammatory cytokine interleukin-10 (IL-10) was upregulated 2.4-fold by SIM-A9s following LnASC co-culture. This increase was not evident in the ObASC group and was significantly less than the expression of the LnASC group. Examination of pro-inflammatory genes yielded a significant increase in expression of iNOS (52-fold), IL-1β (8.5-fold), and tumor necrosis factor alpha (TNFα) (2.8-fold) in ObASC co-cultures, and a non-significant decrease in expression of these genes in LnASC co-cultures. In all three pro-inflammatory genes examined, there was also a significant difference in expression level between the LnASC and ObASC groups. A Griess assay was employed to examine the nitrite concentration of SIM-A9 conditioned media following co-culture (Figure 4D). In agreement with the RAW264.7 data, the ObASC co-cultured SIM-A9 microglia produced 2.7-fold higher levels of nitrite species than untreated controls. This was increased to a 4.6-fold difference when comparing LnASC to ObASC co-cultured SIM-A9 cells.

### 3.5. LnASCs and ObASCs Differentially Affect the Migration and Phagocytic Abilities of SIM-A9 Microglia

**Figure 5:**
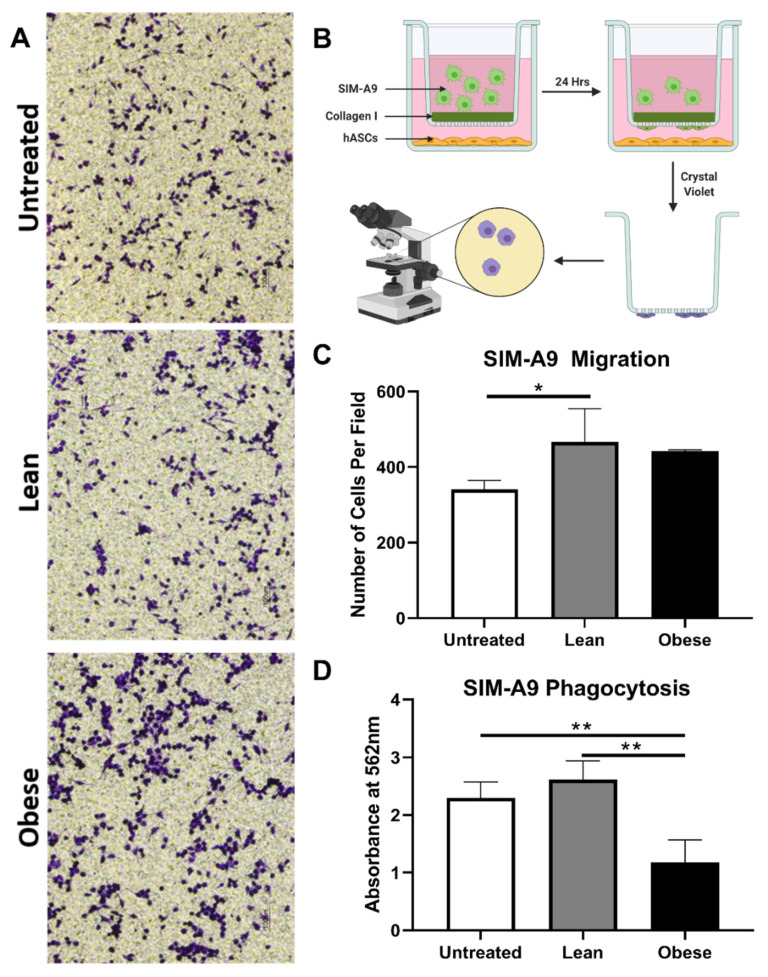
LnASCs and ObASCs differentially alter the migration and phagocytic abilities of SIM-A9 cells. (**A**) Representative images of crystal violet stained SIM-A9 cells following migration through a collagen-coated Transwell in the presence of LnASCs or ObASCs. (**B**) Representative scheme of migration assay process including staining and imaging. Created with Biorender.com (**C**) Quantification of the cells migrated per field based on three randomly chosen fields for each Transwell migration performed. (**D**) Phagocytosis of *E. coli*-substrate by SIM-A9 cells following 48-h treatment with LnASC or ObASC conditioned media. Values are presented as means (N = 3) of three independent experiments ± SD using one-way ANOVA followed by Dunnett’s multiple comparison test. Statistical differences between the means are marked with * *p* < 0.05, ** *p* < 0.01.

Changes in the functional properties of microglia following ASC co-culture were determined. The migration efficiency of SIM-A9 cells was examined using the aforementioned collagen-coated 5 μm pore Transwells. After 24 h, 37% more microglia had migrated in the LnASC co-culture than the untreated controls. ObASC co-cultured SIM-A9s demonstrated an increased trend in migration but failed to reach significance (Figure 5C). Comparisons between the LnASC and ObASC groups also failed to reach significance. The ability of SIM-A9 cells to phagocytose *E. coli* following exposure to LnASC or ObASC conditioned media was also examined (Figure 5D). Exposure to ObASC CM resulted in a 48% decrease in the phagocytic abilities of SIM-A9 cells relative to untreated controls and a 65% decrease relative to LnASC CM exposure.

## 4. Discussion

Adipose stem cells have tremendous therapeutic potential because of their remarkable ability to migrate to sites of inflammation where they recruit immune cells and orchestrate tissue repair [56,57,58]. Adipose stem cells are isolated from donor tissue following liposuction or lipectomy and, as such, are often from obese adipose tissue. Obese adipose tissue is chronically inflamed and hypoxic; therefore, the resident ASCs may be fundamentally altered relative to ASCs from lean donor tissue. Our initial characterization of stemness traits of ObASCs relative to LnASCs yielded results in agreement with numerous previous studies including: reduced adipogenic differentiation capacity [33,59,60,61]; impaired self-renewal ability [36,38]; and diminished expression of standard stem cell phenotypic markers [36,60]. Additionally, our group’s previous work demonstrated an exaggerated immune response of ObASCs to inflammatory stimuli [26], suggesting that the chronic inflammatory environment of obese adipose tissue may alter not only ASC phenotype but also their immunosuppressive function. In support of this theory, an in vivo study conducted by our lab demonstrated a loss of therapeutic efficacy of ObASCs in a mouse model of MS as demonstrated by the lack of symptomatic improvement, lack of lesion reduction, and increased in pro-inflammatory cytokine expression [26]. Therefore, we hypothesized that the ability of ObASCs to induce an anti-inflammatory, pro-repair phenotype in macrophages and microglia would be impaired relative to LnASCs. In the present study, we examined, in vitro, the gene expression profiles, migration, and phagocytic abilities of both macrophages and microglia in the presence of ASCs. We concluded that exposure to ObASCs, but not LnASCs, resulted in pro-inflammatory phenotypes in both macrophages and microglia.

Pro-inflammatory macrophages and microglia exhibit unique gene expression profiles, in part characterized by high levels of iNOS and the production of several cytokines, including IL-1β, IL-6, and TNFα [62]. The secretion of these cytokines results in a pro-inflammatory microenvironment that initiates the polarization of other macrophages and the recruitment and differentiation of naïve T cells toward pro-inflammatory phenotypes. In the present study, we demonstrated that, following co-culture with ObASCs, macrophages significantly upregulated iNOS and IL-1β gene expression when compared with LnASCs and untreated controls. Similarly, microglia exhibited significant upregulation of iNOS, IL-1β, and TNFα transcripts. In contrast, the co-culture of microglia and macrophages with LnASCs demonstrated no significant changes in the expression of pro-inflammatory cytokines in comparison to untreated controls. Direct comparisons made between LnASC and ObASC-exposed cells demonstrated similar significant upregulation of pro-inflammatory genes in the ObASC group. Nitric oxide (NO), the product of iNOS enzymatic activity on L-arginine, is one of the effector molecules of pro-inflammatory phagocytes. It produces metabolites that can be examined as an indirect measure of NO activity. In both cell lines, there were significantly more nitrite species produced following incubation with ObASCs indicating enhanced activity of NO in ObASC exposed cells. No change in NO activity was observed in cells co-cultured with LnASCs. These data suggest that ObASCs, but not LnASCs, promote gene expression resembling the pro-inflammatory polarization of macrophages and microglia in vitro. Interestingly, although the ObASC-exposed SIM-A9 cells significantly downregulated their expression of the anti-inflammatory gene Mrc1, they maintained an elevated expression of Arg1 and similar levels of IL-10 expression as control cells. In addition to being a traditional anti-inflammatory cytokine, IL-10 also has significant effects on the adaptive immune response by promoting the development of regulatory T-cell populations [42]. This combined pro-inflammatory, regulatory microglial phenotype is an illustration of the diversity of potential responses to a complex extracellular signaling milieux. Further, it illustrates the inability of an oversimplified characterization system to appropriately describe these responses.

Gene expression analysis tells an incomplete story and does not demonstrate the effect that ASCs have on the function of macrophages and microglia. As immune surveillance and effector cells, macrophages and microglia must be able to migrate to areas of damage and phagocytose invading pathogens and cell debris. Anti-inflammatory, pro-repair phenotypes express an array of anti-inflammatory cytokines and are responsible for the phagocytosis of cellular debris and orchestration of tissue repair. Our migration assay demonstrated a significant increase in migration of both macrophages and microglia in the presence of LnASCs, which was absent in the presence of ObASCs. These findings are in agreement with several in vitro studies that demonstrated increased speed and distance of migration in a synthetic extracellular matrix by both M2 macrophages and microglia relative to M1 macrophages and microglia [63,64]. Similar results were also found in a rat microglial migration study, which noted significant M2 microglial migration through a matrix relative to M1 [65]. Phagocytosis is another function of macrophages and microglia and is essential for pathogen defense and tissue regeneration. Previous studies have demonstrated a decrease in the ability of macrophages to phagocytose E. coli following polarization to M1 [66,67]. Similarly, our results demonstrated decreased phagocytosis in the macrophages and microglia exposed to ObASCs. The cells that were exposed to LnASCs exhibited phagocytic abilities very similar to untreated control cells. Taken together, the decrease in mobility and phagocytic abilities of both macrophages and microglia are suggestive of polarization to an M1 phenotype as a result of exposure to ObASCs. Persistent M1 polarization results in a pro-inflammatory extracellular milieux which is essential for fighting infection and recruitment of additional immune cell populations, but it can also result in extensive tissue damage. M2 polarization is important for the reduction of inflammation, degradation of cellular debris, and initiation of tissue repair mechanisms. Thus, ObASCs may possess limited therapeutic potential because of their promotion of pro-inflammatory phenotypes.

The data from these studies suggest that ObASCs are fundamentally altered by their environment. This alteration manifests as a phenotype that preferentially induces a pro-inflammatory polarization in both macrophages and microglia, as evidenced by elevation of pro-inflammatory transcripts, decreased mobility, and diminished phagocytosis. The results of this study illustrate the importance of understanding the impact of the tissue niche on ASC phenotype and immunomodulatory function. In the context of an inflammatory disease such as MS, the skewed immunomodulatory function of ObASCs which promotes pro-inflammatory activation of innate immune cells may help explain the lack of therapeutic effect in previous studies. However, MS is only one of the numerous potential disease applications for ASCs, each of which are defined by unique pathogenic mechanisms. An ASC therapeutic ideally functions as a biological immunomodulator. One that enhances the pro-inflammatory function of macrophages or microglia may be beneficial for situations in which normal immune function is diminished or compromised. Successful translation of ASC therapeutics to the clinic will require the selection of ASCs that best fit the desired immunomodulatory response and produce the intended therapeutic outcome.

## Figures and Tables

**Table 1:** Primer Sequences.

Target Gene	Forward	Reverse
iNOS	5′-GCCACCAACAATGGCAACA-3′	5′-CGTACCGGATGAGCTGTGAATT-3′
IL-1β	5′-CCTGCAGCTGGAGAGTGTGGAT-3′	5′-TGTGCTCTGCTTGTGAGGTGCT-3′
TNFα	5′-ATGGCCTCCCTCTCATCAGTTC-3′	5′-TTGGTGGTTTGCTACGACGTG-3′
Arg1	5′-GTGAAGAACCCACGGTCTGT-3′	5′-CCAGCACCACACTGACTCTT-3′
Mrc1	5′-GTGGAGTGATGGAACCCCAG-3′	5′-CTGTCCGCCCAGTATCCATC-3′
IL-10	5′-GCTCTTGCACTACCAAAGCC-3′	5′-CTGCTGATCCTCATGCCAGT-3′
RPS29	5′-TTCCTTTCTCCTCGTTGGGC-3′	5′-TTCAGCCCGTATTTGCGGAT-3’

## Data Availability

The data presented in this study are available on request from the corresponding author. The data are not publicly available.
